# Fabrication and Characterization of Poly(vinyl alcohol)-chitosan-capped Silver Nanoparticle Hybrid Membranes for Pervaporation Dehydration of Ethanol

**DOI:** 10.3390/gels8070401

**Published:** 2022-06-24

**Authors:** Manu L. Naik, Ashok M. Sajjan, T. M. Yunus Khan, Ashwini M, Sharanappa Achappa, Nagaraj R. Banapurmath, Narasimha H. Ayachit, Mostafa A. H. Abdelmohimen

**Affiliations:** 1 Department of Chemistry, KLE Technological University, Hubballi 580031, India; manunaik@kletech.ac.in; 2 Center for Material Science, KLE Technological University, Hubballi 580031, India; nr_banapurmath@kletech.ac.in (N.R.B.); ayachit@kletech.ac.in (N.H.A.); 3 Department of Mechanical Engineering, College of Engineering, King Khalid University, Abha 61421, Saudi Arabia; mtatagar@kku.edu.sa (T.M.Y.K.); mmhussien@kku.edu.sa (M.A.H.A.); 4 AICRP on EAAI (Bioconversion Technology) MARS, University of Agricultural Sciences, Dharwad 580005, India; ashwinim21@gmail.com; 5 Department of Biotechnology, KLE Technological University, Hubballi 580031, India; sharanappaa@kletech.ac.in; 6 Shoubra Faculty of Engineering, Benha University, Cairo 11629, Egypt

**Keywords:** permeation flux, selectivity, hydrophilicity, amorphous, crystallinity

## Abstract

Chitosan-capped silver nanoparticle (CS-capped AgNPs)-incorporated Poly(vinyl alcohol) (PVA) hybrid membranes were prepared by a solution-casting technique for ethanol dehydration via pervaporation. The incorporation of CS-capped AgNPs into the PVA membrane and its influence on membrane properties and pervaporation-separation process of azeotropic water/ethanol mixture was studied. The addition of CS-capped AgNPs into the PVA membrane reduced the crystallinity, thereby increasing the hydrophilicity and swelling degree of the hybrid membrane, supported by contact angle (CA) analyzer and swelling degree experiments, respectively. Fourier transform infrared spectroscopy (FTIR) demonstrated the formation of polymeric matrix between PVA and CS and also the binding of AgNPs onto the functional group of CS and PVA, which was also reflected in the microstructure images demonstrated by scanning electron microscopy (SEM) and by 2θ angle of wide-angle X-ray diffraction (WAXD). The effect of CS-capped AgNPs on the thermal stability of the hybrid membrane was demonstrated by differential scanning calorimetry (DSC) and thermogravimetric analyzer (TGA). These characteristics of the hybrid membrane positively impact the efficiency of the dehydration of ethanol, as indicated by pervaporation experiments. The best performances in total flux (12.40 ± 0.20 × 10^−2^ kg/m^2^ h) and selectivity (3612.33 ± 6.03) at 30 °C were shown for CS-capped AgNPs PVA hybrid membrane containing 2 wt.% CS-capped AgNPs (M-4). This confirms that the developed hybrid membranes can be efficiently used to separate water from azeotropic aqueous ethanol.

## 1. Introduction

Ethanol is a versatile natural solvent and is miscible in water and many other organic solvents. [1,2]. Ethanol is produced mostly via fermentation process through the action of microorganisms by using corn or sugar-cane molasses as substrate [3,4,5]. It is used in the medical field as an antiseptic agent [6] and principally as fuel, as a substitute for petroleum in road-transport vehicles [7]. The ethanol produced by fermentation can be purified by extractive distillation, azeotropic distillation, adsorption, liquid–liquid extraction, and crystallization, which are often inefficient and uneconomical [8,9]. Ethanol separation from the azeotropic composition, which consists of 95.6% (*v*/*v*) concentration of ethanol and 4.4% (*v*/*v*) of water, is carried out by azeotropic distillation, which is an energy-intensive process with a large capital cost and health and safety concerns [10,11]. Instead, the petrochemical and biochemical industries consider pervaporation a potential candidate for separating ethanol from its azeotropic composition. [12].

Pervaporation (PV), in its simplest type, is a combination of membrane permeation and evaporation associated with lower energy consumption and an economical process [13,14,15]. PV is a membrane-separation process that uses a dense polymeric membrane to selectively permeate one or more components from a liquid mixture. In the process, the liquid mixture is kept in direct contact with one side of the membrane, and the permeated product (pervaporate) is removed in the form of vapor and later condensed. The productivity and success of the pervaporation process largely depend on the fabrication of suitable membranes with high permeability, good selectivity and sufficient mechanical strength [16]. In particular, hydrophilic groups containing new membrane materials are preferred for the dehydration of alcohols. The membranes mainly considered in the PV process for dehydration of alcohols are Poly(vinyl alcohol), sodium alginate and chitosan due to their excellent hydrophilicity and good film-forming ability [17,18,19,20].

In the PV process, Poly(vinyl alcohol) (PVA) is a commonly used polymer for developing membranes. Advantages of using PVA membranes in the PV process are their non-toxic nature, water solubility due to the presence of the hydroxyl group and being biocompatible and biodegradable synthetic polymers [21,22,23]. The PVA has a high affinity towards water due to the formation of hydrogen bonds between intra- and intermolecular hydroxyl groups [19]. The separation performance of PVA membranes is unsatisfactory due to the swelling phenomenon in the presence of water, high crystallinity restricting from intermolecular hydrogen bonding, and instability. The PVA membrane has been modified to overcome this problem through cross-linking, filling, blending and grafting [24,25,26,27,28]. Despite this, the expected performance was not able to be achieved.

On the contrary, hybrid materials combine the important properties of both inorganic and organic and create new compositions with unique characteristics, which offer specific advantages for fabricating PV membranes exhibiting high selectivity and flux with desirable thermal and chemical strength. Perhaps, this becomes a facile and feasible way to solve the trade-off phenomenon, which generally exists in the PV process [29,30,31].

Considering this, Wang et al. [32] developed PVA hybrid membranes containing different graphite carbon-nitride nanosheets to dehydrate ethanol by PV. They achieved a separation factor of 57.9 and a total flux of 2.328 kg.m^−2^ h^−1^. Jiang et al. [33] had overcome the trade-off phenomena with organic and inorganic hybrid membranes by incorporating graphite into the PVA matrix and increased both flux (0.0913 kg.m^−2^ h^−1^) and selectivity (92). Xia et al. [34] developed PVA nanohybrid membranes by incorporating organosilica, and used this membrane for ethanol dehydration by PV and achieved selectivity of 1026 and flux of 0.145 kg.m^−2^ h^−1^. Our previous work [35] fabricated B_2_SA-grafted Poly(vinyl alcohol)–graphene hybrid membranes for ethanol dehydration by PV. Among these, the membrane containing 2 wt.% of graphene exhibited selectivity of 4187 with a flux of 0.1166 kg.m^−2^ h^−1^.

Chitosan is the most abundant biopolymer and is deacetylated form of chitin. It exhibits hydrophilicity, degradation and antibacterial properties. It can adsorb metal ions using chelation due to the lone pair of electrons in its amino nitrogen [36,37,38,39,40,41]. It has better compatibility with PVA due to its hydrogen-bonding interaction [41].

Silver nanoparticles (AgNPs) are used in different fields, including medical, food, health care, consumer and industrial fields, due to their unique physical and chemical properties. These include optical, electrical and thermal properties, high electrical conductivity, biological properties [42,43,44] and hydrophilic nature [45].

In the present investigation, by understanding the pros and cons of developing hybrid membranes, we attempted to develop hybrid membranes by suitably incorporating CS-capped AgNPs. Different characterization methods are used to examine the physical and chemical properties of the fabricated membranes. Effects of CS-capped AgNPs and temperature on PV performance were systematically studied. The performance of fabricated membranes was evaluated by correlating with their structural properties.

## 2. Results

### 2.1. Membrane Characterization

#### 2.1.1. FTIR Studies

**Figure 1:**
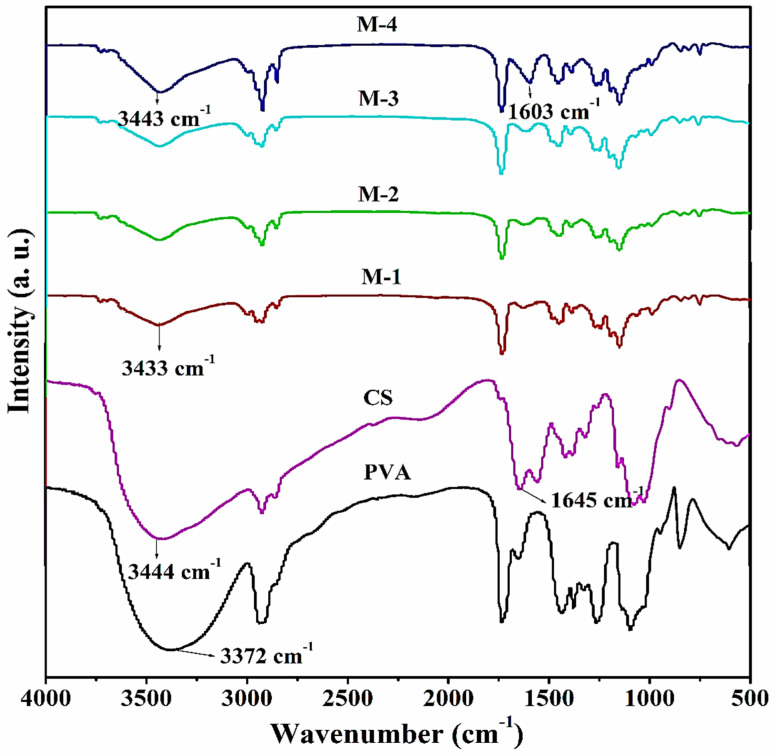
FTIR spectra of pure PVA, Chitosan, CS-capped AgNPs incorporated hybrid PVA membranes: (M-1) 0.5 wt.%; (M-2) 1 wt.%; (M-3) 1.5 wt.%; (M-4) 2 wt.% of CS-capped AgNPs.

Figure 1 shows the FTIR spectra of PVA, CS and CS-capped AgNPs incorporated in PVA membranes; the O-H stretching is visible with a broad peak at 3372 cm^−1^ in the FTIR spectra of the PVA membrane, and multiple brands appeared between 1000 to 1200 cm^−1^ corresponding to –C-O stretching vibrations of the hydroxyl groups. These bands matched those reported by Sajjan et al. [46]. The wide visible peak establishes the occurrence of the hydroxyl functional group at 3444 cm^−1^, and bands appeared at around 1645 and 1550 cm^−1^ corresponding to NH (amide I) and amide II functional groups of the pure chitosan [47,48].

In the case of CS-capped AgNPs incorporated hybrid PVA membranes, broadband appeared at 3433 cm^−1^, showing the movement towards the higher wavenumber along with the increase in the intensity of the band as we progressed from M-1 (0.5% CS-capped AgNPs) to M-4 (2 wt.% CS-capped AgNPs), indicating enhanced hydrogen bonding due to PVA-CS chain interactions [49]. Multiple peaks owing to various amide functional groups that showed in pure chitosan are absent in membranes M-1 (0.5% CS-capped AgNPs) to M-4 (2 wt.% CS-capped AgNPs), demonstrating that these functional groups are involved in AgNP stability. At 1603 cm^−1^, a new peak appeared. Its peak intensity increased as the wt.% of CS-capped AgNPs increased in the PVA matrix, indicating that silver particles are bound to the functional groups present in chitosan and PVA. The shifting of the peak is due to the formation of a coordination bond between the silver atom and the electron-rich group present in PVA and chitosan [47,49]

#### 2.1.2. Wide-Angle X-ray Diffraction Studies (WAXD)

**Figure 2:**
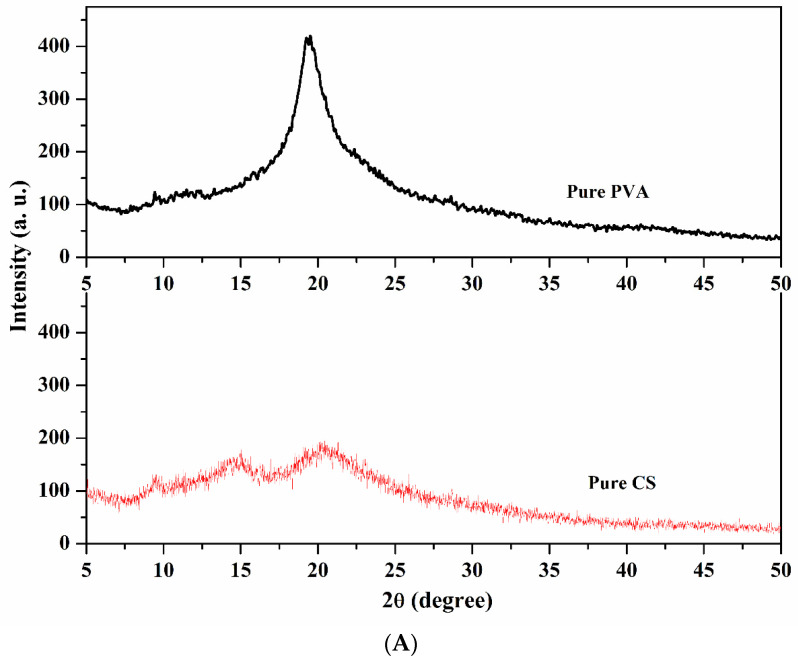

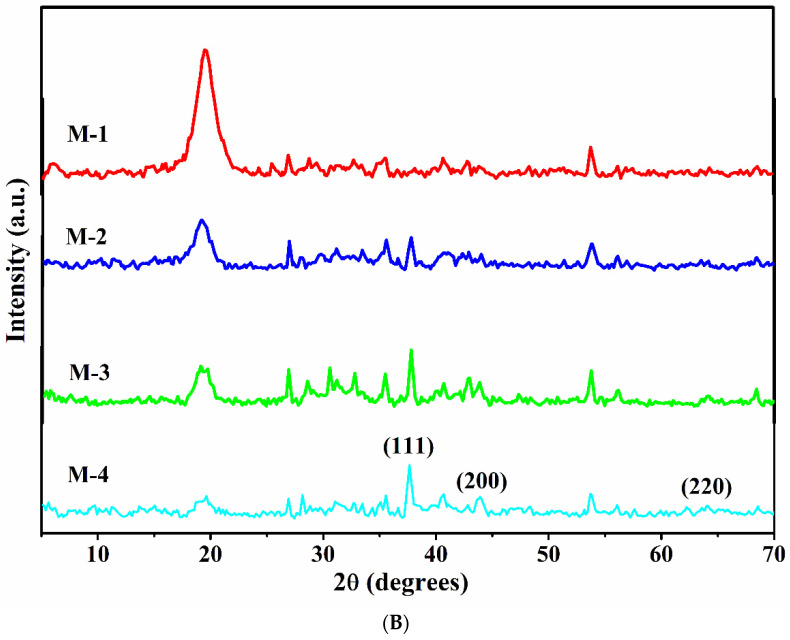
(**A**). Wide-angle X-ray diffraction patterns of plane PVA and Chitosan. (**B**). Wide-angle X-ray diffraction patterns of CS-capped AgNP-incorporated PVA membranes: (M-1) 0.5 wt.%; (M-2) 1 wt.%; (M-3) 1.5 wt.%; (M-4) 2 wt.% of CS-capped AgNPs.

The illustrations of the WAXD pattern of PVA and CS-capped AgNPs embedded in PVA membranes are illustrated in Figure 2A,B. The plane PVA membrane exhibited a peak at 2*θ* = 20°, which indicates the presence of crystalline and amorphous regions in the morphology of the membrane [46]. The diffraction-peak intensity of PVA was reduced as the content of CS-capped AgNPs in the PVA matrix increased. This is due to the CS-capped AgNPs, which disorient the PVA chains and enhance the amorphous regions by reducing the crystalline regions. Further, the peak of the M-2 membrane was weaker than that of the PVA membrane, representing strong interaction among PVA and CS-capped NPs. Thus, the XRD results align with the FTIR results, in that some interaction occurred between PVA and CS-capped AgNPs. It implies that the PVA and CS-capped AgNP matrix presents high compatibility with stronger interactions [49]. The peaks that appeared at 2θ = 27°, 38° and 55° also confirm the presence of AgNPs. All this evidence suggests that an amorphous nature is predominant in the membranes where CS-capped AgNPs content is present, which supports the selective movement of the molecules through the membranes.

#### 2.1.3. Thermogravimetric Analysis (TGA)

**Figure 3:**
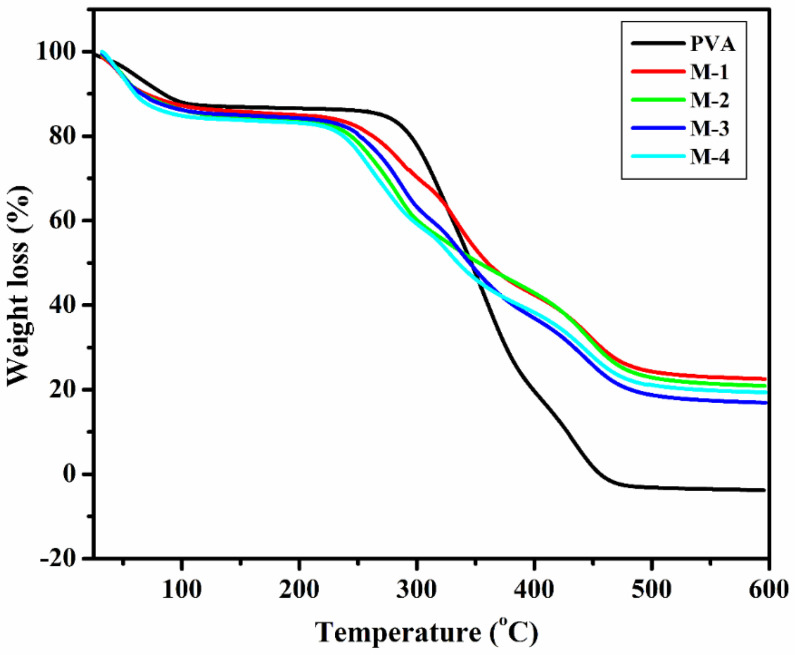
Thermogravimetric analysis patterns of PVA, CS-capped AgNP-incorporated PVA hybrid membranes: (M-1) 0.5 wt.%; (M-2) 1 wt.%; (M-3) 1.5 wt.%; (M-4) 2 wt.% of CS-capped AgNPs.

Figure 3 exhibits the obtained thermogram of the produced membranes. Thermal degradation of the membranes took place in two phases for all the membranes. Weight loss occurred between ambient temperature and 125 °C, corresponding to the volatilization of water and other volatile molecules in the membrane. The absorbed water molecule exists in the bound rather than the free molecular state [50]. Such a weight loss is about 12% for the PVA membrane, whereas CS-capped AgNP-incorporated PVA hybrid membranes exhibited a higher loss ranging from 17–18%, due to higher water-retention capacity.

Further, incorporated CS-capped AgNPs disturbed the PVA chains due to the intermolecular hydrogen bonding between PVA and CS-capped AgNPs and electrostatic interaction with AgNPs, responsible for decreased crystallinity of the PVA membrane; hence, CS-capped AgNPs membranes have higher water-retention capacity. The second stage of decomposition of the PVA membrane starts from 283–465 °C, attributed to major weight loss due to the decomposition of polymer chains [51]. Compared with PVA in the second stage, the CS-capped AgNP-incorporated PVA hybrid membranes showed 32–35 °C lower than the PVA membrane, which indicates that CS-capped AgNP-incorporated membranes demonstrated lower degradation temperature due to hydrogen bonding and decreased crystallinity. This is well-supported by FTIR and WXRD studies. The CS-capped AgNP-incorporated PVA hybrid membrane demonstrated better thermal resistance since their residue is larger than the PVA membrane.

#### 2.1.4. Differential Scanning Colorimetry (DSC)

**Figure 4:**
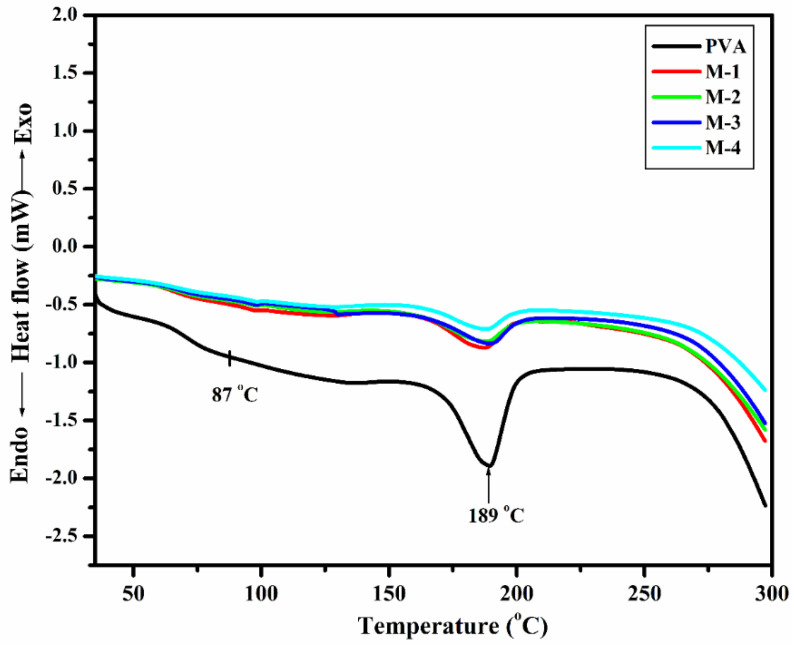
DSC thermograms of PVA and CS-capped AgNP-incorporated PVA hybrid membranes: (M-1) 0.5 wt.%; (M-2) 1 wt.%; (M-3) 1.5 wt.%; (M-4) 2 wt.% of CS-capped AgNPs.

The glass-transition-temperature (T_g_) investigation was performed to characterize the thermal behavior of the produced membranes. The membrane thermograms (Figure 4) were obtained using DSC analysis at temperatures ranging from 27 to 250 °C. The T_g_ of the pure PVA membrane, according to these thermograms, was around 85 °C [46]. T_g_ was then gradually increased from 90 to 101 °C as the amount of CS-capped AgNPs in the PVA matrix grew from M-1 (0.5 wt.% CS-capped AgNPs) to M-4 (2 wt.% CS-capped AgNPs). The interaction between PVA and CS-capped AgNPs causes chain mobility to be hampered, resulting in an increase in T_g_. When the proportion of CS-capped AgNPs in the PVA membrane matrix increased, the T_g_ values increased from M-1 (0.5 wt.% CS-capped AgNPs) to M-4 (2 wt.% CS-capped AgNPs), indicating that the PVA membranes became more stable.

#### 2.1.5. Scanning Electron Microscopy (SEM)

**Figure 5:**
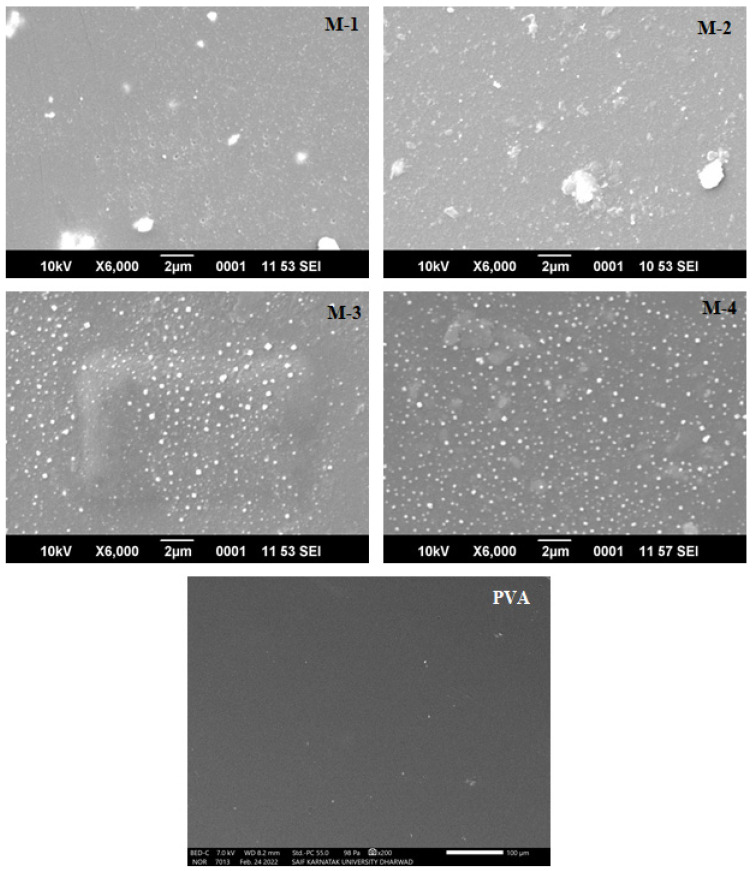
SEM micrographs of PVA and CS-capped AgNP-incorporated PVA hybrid membranes: (M-1) 0.5 wt.%; (M-2) 1 wt.%; (M-3) 1.5 wt.%; (M-4) 2 wt.% of CS-capped AgNPs.

SEM analysis of the fabricated membranes was carried out to investigate the effect of CS-capped AgNPs on the microstructure of the membranes, and the resulting micrographs are shown in Figure 5. The bright patches in the membrane were enhanced as we progressed from M-1 (0.5 wt.% CS-capped AgNPs) to M-4 (2 wt.% CS-capped AgNPs), owing to an increase in the CS-capped AgNP content in the membrane. There was no cluster formation in the membrane matrix when the CS-capped AgNPs were distributed. These images also showed that all of the CS-capped AgNPs were disseminated in the membrane independently and that the membranes were homogeneous and defect-free. Furthermore, these photos demonstrated that the PVA matrix and CS-capped AgNPs are compatible.

#### 2.1.6. Contact-Angle Analysis

**Figure 6:**
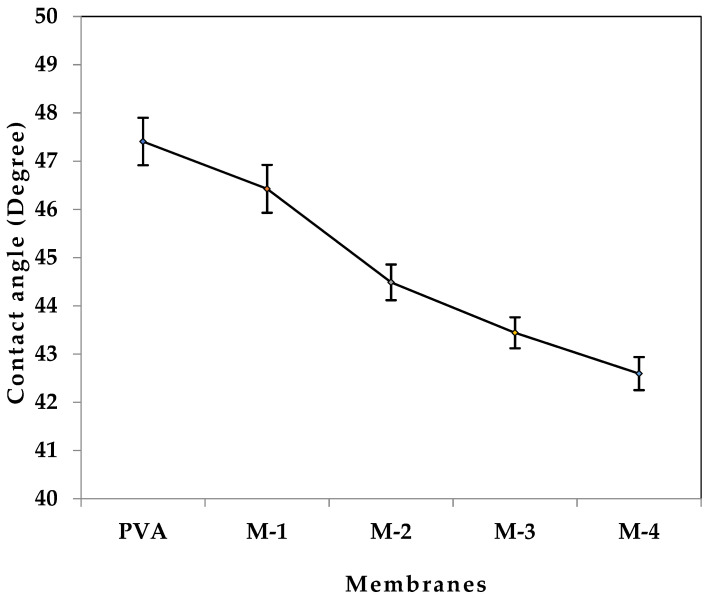
Contact angles of water for all the membranes at 10 s.

Figure 6 shows the contact angles of PVA and CS-capped AgNPs embedded into PVA membranes. The contact angle of all the membranes was decreased with the increase in the CS-capped AgNP content in the PVA matrix. This demonstrated that incorporating CS-capped AgNPs in the PVA membrane increased the hydrophilicity of the hybrid membrane. This was due to the increased amount of different hydrophilic groups provided by the CS-capped AgNPs, mainly –OH, –NH and AgNPs. Moreover, the disturbance in the regularity of the PVA matrix structure results in lowering the crystallinity of the PVA and hydrogen-bonding network, thus enhancing the availability of the pre–OH groups in the membrane matrix. An increase in the hydrophilicity and decrease in crystallinity of the membrane increases the affinity for the water molecules. As a result, the developed membrane is more effective for pervaporation, especially for the dehydration of low-water-content ethanol [52]. The sorption analysis reflects these findings as well. This illustrates the membrane’s improved water affinity with enhancement in the CS-capped AgNPs in the PVA membrane matrix.

### 2.2. Effect of Amount of CS-Capped AgNPs on Membrane Swelling

**Figure 7:**
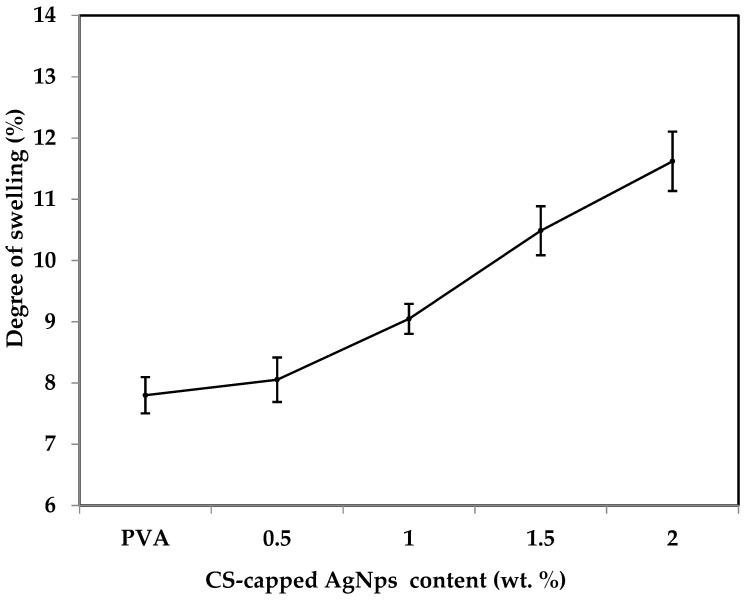
Effect of *CS-capped AgNPs* on membrane swelling degree.

Figure 7 shows the observed swelling level of the membranes. From the figure, the extent of swelling increased from membrane M-1 (0.5 wt.% CS-capped AgNPs) to M-4 (2 wt.% CS-capped AgNPs). This is because of the increased amount of different hydrophilic groups provided by the CS-capped AgNPs, mainly –OH, –NH and AgNPs in the PVA membrane matrix, which has a hydrophilic property. This leads to a higher affinity between the fabricated hybrid membranes and the H_2_O molecules, and the percentage of the degree of swelling enhances. This enhanced hydrophilic nature of the fabricated hybrid membranes increases the efficiency of PV performance. These results are in line with the contact-angle results.

### 2.3. Effect of Amount of CS-Capped AgNPs on Pervaporation

Table 1 shows the findings on the pervaporation performance of the fabricated hybrid membranes at various temperatures. As shown in the table, as the quantity of CS-capped AgNPs in the fabricated hybrid membrane increased, the separation selectivity, total permeation flux and permeance were also enhanced. In sorption studies, a similar pattern of increase was seen. The increase in total permeation flux was systematic. The M-4 exhibited the highest total permeation flux of 0.1240 ± 0.0020 kg/m^2^ h at 30 °C. According to these results, the addition of CS-capped AgNPs improves the membranes’ water affinity. AgNPs are responsible for increased polar nature and decreased crystallinity, which enhances the mechanism of diffusion through the membrane.

The membrane’s separation selectivity is mostly determined by the molecular size and free volume of the polymer-membrane matrix. The data revealed that separation selectivity was improved as the concentration of CS-capped AgNPs was increased. This is due to the increased amorphous regions in the membrane, which pilots the enhancement of the free volume of the polymer chains. Because of this, the diffusion of the water becomes easier compared to ethanol, owing to its smaller size. Further, polar groups such as **–**OH and **–**NH_2_ present in PVA and chitosan, respectively, leading to the significant enhancement of hydrogen bonding and electrostatic force of attraction and makes the diffusion of water easier compared to the diffusion of ethanol. Because of the trade-off phenomenon, it is uncommon to see improvements in separation selectivity and total permeation flux. However, this work saw antitrade-off phenomena when CS-capped AgNPs were added to the PVA membrane matrix. A significant enhancement of both hydrophilicity and the amorphous nature of the membrane by incorporating CS-capped AgNPs overcame the trade-off phenomena.

Two-way ANOVA with replication was performed at a 95% (α = 0.05) confidence level to see the effect of membrane type and temperature on permeation flux and selectivity, as represented in Table 2. Based on the *p*-value for membrane type (*p* = 9.29 × 10^−29^ & 2.23 × 10^−58^ < 0.05 = α), temperature (*p* = 1.76 × 10^−14^ & 2.46 × 10^−48^ < 0.05 = α), an interaction effect between membrane type and temperature (*p* = 0.0432 & 6.46 × 10^−40^ < 0.05 = α) shows that the factors are statistically significant by rejecting the null hypothesis. To see the difference in means of the parameters between membrane type and temperature, a post hoc test using Tukey’s HSD was performed after the two-way ANOVA. A Tukey test on membrane type showed a significant effect on total permeation flux and selectivity, as means between all membranes were higher than the mean critical of 0.002576 for total permeation flux and 9.3161 for selectivity, respectively. Similarly, temperature showed a significant effect on total permeation flux and selectivity, as means between all temperatures were higher than the mean critical of 0.001696 for total permeation flux and 6.132 for selectivity, respectively. However, the Tukey test showed no significant effect between membrane type and temperature for total permeation flux and selectivity.

**Figure 8:**
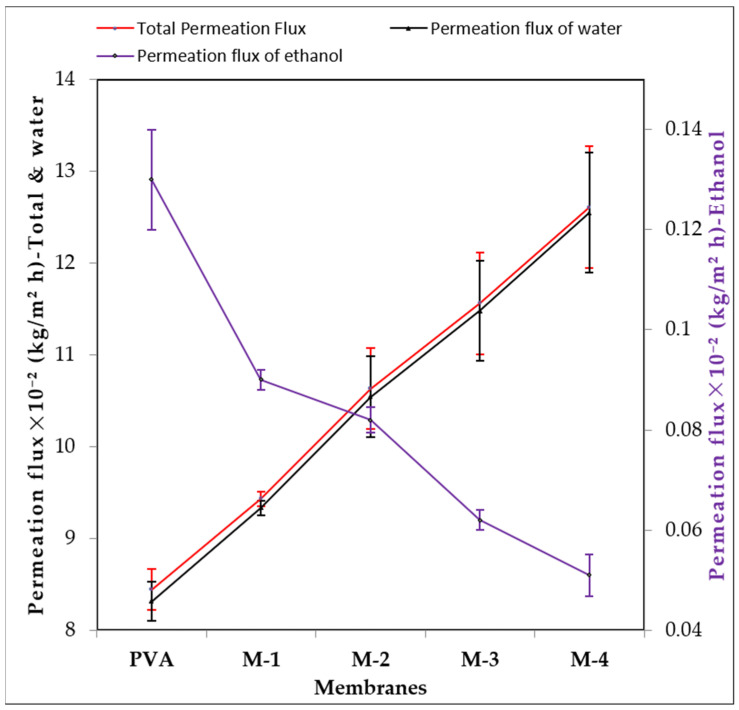
Deviation in total permeation flux and permeation fluxes of water and ethanol at 30 °C.

Figure 8 shows ethanol, water and total permeation flux as a function of CS-capped AgNP content. The graph clearly shows that the permeation flux curves of total and water overlap very closely. Ethanol, on the other hand, has a low permeation flux. The hydrophilicity and amorphous nature of the fabricated hybrid membranes confirm the nature of the curves.

### 2.4. Effect of CS-Capped AgNPs on the Pervaporation-Separation Index (PSI)

**Figure 9:**
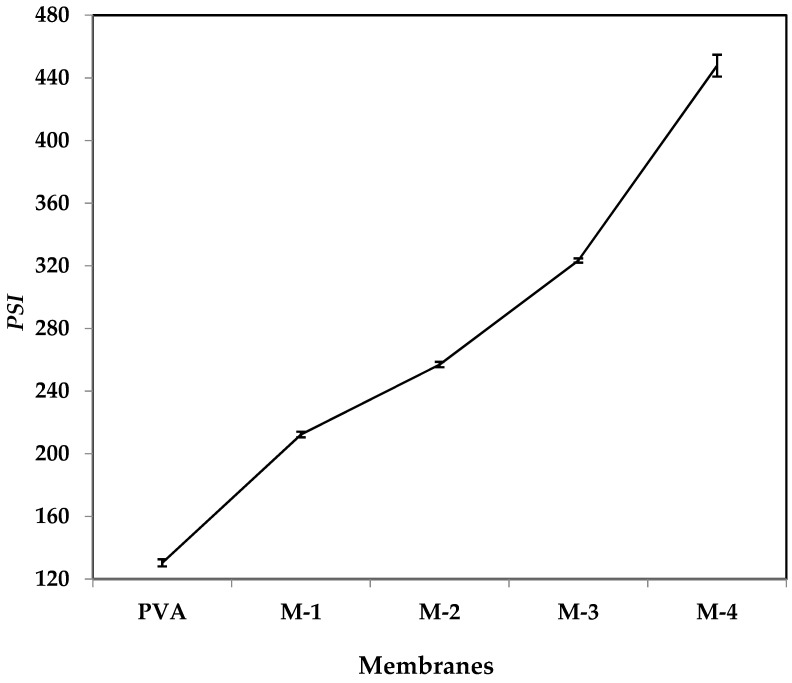
Effect of CS-capped AgNP content on PSI at 30 °C.

The pervaporation-separation index (PSI) is the product of total permeation flux and selectivity, which characterizes the membrane-separation ability. This index can be used as a relative guideline for designing new membranes for the pervaporation-separation process and selecting a membrane with an optimal combination of flux and selectivity. To assess the overall performance, the calculated PSI data are plotted as a function of wt.% of CS-capped AgNPs for the azeotropic water-ethanol mixture (Figure 9). It is observed that the PSI values were increased regularly with increasing the CS-capped AgNPs content, signifying that the membranes with a higher amount of CS-capped AgNPs exhibited excellent performance for separation of the azeotropic water-ethanol mixture. This is attributed to the incorporation of CS-capped AgNPs into the PVA membrane matrix, which changes the hydrophilicity of the membranes and their amorphous nature, significantly influencing the diffusion process. Sorption is only the first step, but in the second step of diffusion, the properties of CS-capped AgNPs played a major role in enhancing the overall performance of the membrane.

### 2.5. Effect of Temperature on Membrane Performance

The effect of operating temperature on the PV performance for the azeotropic water-ethanol mixture was studied for all the membranes, and the resulting values are presented in Table 1. It is observed that the permeation rate was found to increase from 30 to 50 °C for all the membranes while decreasing the separation factor noticeably. Higher temperature decreases the intermolecular interaction between permeants and decreases the intermolecular interaction within the membrane material, resulting in increased free –OH and –NH_2_ groups on the membrane. These are responsible for predominating the plasticizing effect on the membrane due to greater swelling. Therefore, the permeation of diffusing molecules and the associated molecules through the membrane becomes easier, increasing total permeation flux while suppressing the selectivity.

## 3. Conclusions

In this study, new hybrid PVA membranes were prepared by incorporating different wt.% of CS-capped AgNPs for applications in ethanol dehydration from azeotropic water/ethanol mixtures. Increased CS-capped AgNPs content in the membrane matrix resulted in simultaneous permeation flux and selectivity increases. This was explained based on the significant enhancement of hydrophilicity and the amorphous nature of the membrane matrix. The PV separation-index data also indicates that the membrane incorporated with 2 wt.% CS-capped AgNPs (M-4) showed an excellent performance while separating the water-ethanol mixtures because of enhanced hydrophilicity and selective interaction between the membranes and permeate. The membrane M-4 showed the highest total flux (12.40 ± 0.20 × 10^−2^ kg/m^2^ h) and selectivity (3612.33 ± 6.03) at 30 °C for 10 mass% of water in the feed. Experimental data also revealed that total flux and water flux are almost overlapping for all the CS-capped AgNP-incorporated membranes, suggesting that the developed membranes have higher separation ability. The temperature effect study indicated that permeation flux was increased while decreasing selectivity when the operating temperature was increased. This is mainly because of decreased viscosity of permeating molecules.

## 4. Materials and Methods

### 4.1. Materials

The chemicals used in the current study are as follows: Poly(vinyl alcohol), PVA (MW = 85–124 kDa, DH = 86 to 89%), and Acetic acid (purity ≥ 99.5%) were purchased from s.d.fine Chemicals Ltd., Mumbai, India. Chitosan, CS (MW = 200 kDa; N-deacetylation degree 75–85%) and silver nanopowder (purity ≥ 99.5%, particle size < 150 nm) were purchased from Sigma-Aldrich Chemicals, St. Louis, MO, USA. Rectified ethanol (95.6 vol.%) from Godavari Biorefineries, Karnataka, India. Chemicals purchased were used without further purifications as they are reagent-grade chemicals.

### 4.2. Preparation of Chitosan-Capped Silver Nanoparticles

The purpose of fabricating CS-capped Ag nanoparticles was to improve the PVA membrane in terms of PV performance. The fabrication procedure was as follows: In 50 mL of water, 2 vol.% acetic acid and 1 g chitosan were added and mixed for 24 h at room temperature. A total of 0.25 g of AgNPs was combined in this homogeneous solution and agitated continuously for 24 h at 60 °C to generate a suspension of CS-capped AgNPs. This dispersion was then sonicated for 30 min at a constant frequency of 38 kHz in an ultrasonic bath (Grant XB6, Shepreth, Cambridgeshire, UK) to break up any possible silver-nanoparticle clumps.

### 4.3. Membrane Preparation

Poly(vinyl alcohol) (4 g) was dissolved in deaerated water (96 mL) and agitated at 30 °C for 24 h. Further, the solution was filtered, and the undissolved particles were removed. The solution was then gently spread out on a glass plate and allowed to dry for 72 h. The membrane was then carefully peeled away and labeled M.

**Figure 10:**
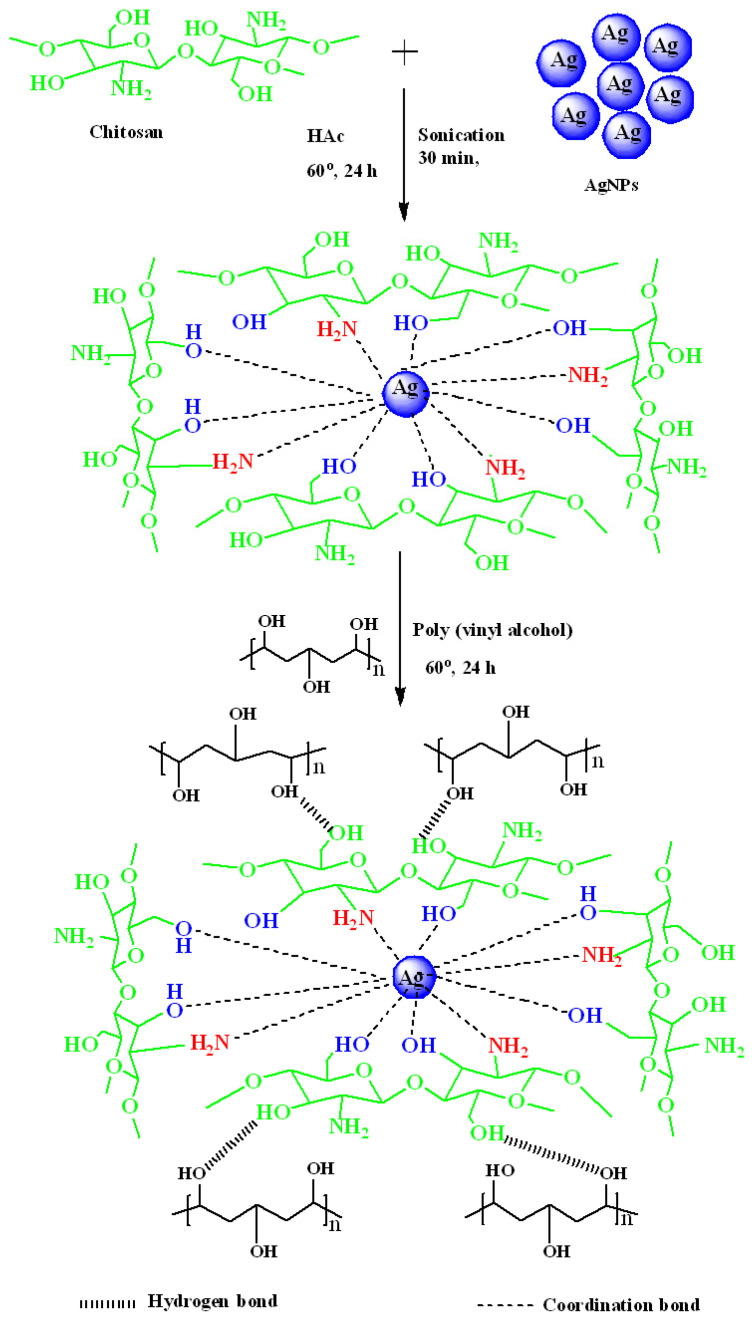
Scheme for the fabrication of CS-capped AgNP-incorporated PVA hybrid membranes.

The development of the CS-capped AgNPs-Poly(vinyl alcohol) membrane was carried out according to the procedure mentioned ahead: To uniform PVA solution, a fixed amount of CS-capped AgNPs suspension was mixed and stirred for 24 h. The prepared mixture was then passed through a sonication process maintained at 40 kHz for 2 h for proper distribution of CS-capped AgNPs in the base matrix (PVA). The prepared solution was cast on a clean glass plate. The rest of the procedure is similar to the procedure followed for the fabrication of M. Known amounts (0.5, 1.0%, 1.5%, 2 wt.%) of CS-capped AgNPs were added, and the obtained membranes were named M-1, M-2, M-3 and M-4. The breadth of the fabricated membranes was measured with a thickness gauge at several locations with a precision of 2 µm. (Peacock dial thickness gauge). A constant thickness of 50 ± 2 µm was achieved. The scheme of the fabrication of CS-capped AgNP-incorporated PVA membrane is shown in Figure 10.

### 4.4. Membrane Characterizations

The structural interactions between CS-capped AgNPs and PVA membrane were studied by Spectrum two with Diamond ATR Fourier transform infrared (FTIR) spectroscopy (PerkinElmer Pvt. Ltd., 28, Ayer Rajah Crescent, Singapore). FTIR analysis was recorded in the range of 500 to 4000 cm^−1^ and with a spectral resolution of 4 cm^−1^.

A wide-angle X-ray diffractometer (Rigaku SmartLab SE, Tokoyo, Japan) was used to identify the solid-state morphology of the membranes developed. Ni-filtered CuKα radiation (30 mA) was used as an X-ray source, and a cathode current of 40 kV was applied. Samples of membranes in the range of 5° to 70° were scanned at the rate of 8°/min for the angle 2θ. Differential scanning calorimetry (DSC) measurements (DSC Q 20, TA Instruments, Waters LLC, New Castle, DE, USA) and Thermogravimetry analyzer (TGA) (DSC Q20, TA Instruments, Waters LLC, New Castle, DE, USA) was used for thermal analysis of the fabricated membranes. Scanning electron microscope (SEM) (JEOL-JSM-IT500, Tokyo, Japan) was used to study the surface morphology of the fabricated membranes.

In order to study the water affinity of the fabricated membranes at 30 °C, the contact angle was measured, employing a contact-angle meter named Acam-series, Apex Instruments Co. Pvt. Ltd., Kolkata, India. In order to further confirm the hydrophilic nature of the developed membranes, membrane-sorption analysis was conducted according to the procedure mentioned in the previous study [16].

### 4.5. Pervaporation Experiments

**Figure 11:**
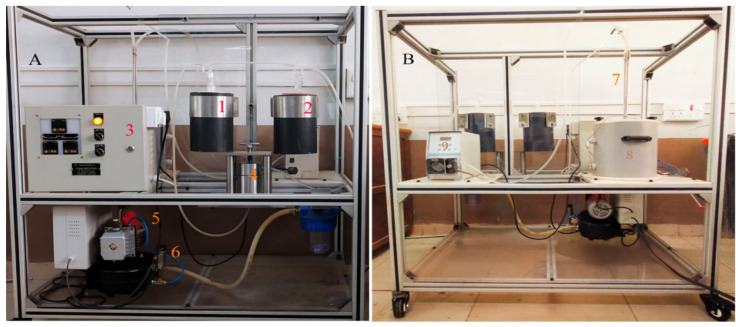
Photographic image of the pervaporation apparatus. (**A**) Front view: (1) permeate cold trap; (2) moisture cold trap; (3) control panel; (4) pervaporation cell; (5) vacuum pump; (6) vacuum control sensor. (**B**) Back view: (7) inlet and outlet of the feed tank; (8) feed tank; (9) circulation pump.

For PV experiments, a specifically designed PV apparatus was used, and the photographs of the PV unit are shown in Figure 11. The apparatus was equipped with a heating vessel of 145 mm in diameter with a volume of 3 L used to fill the azeotropic water-ethanol mixture. The tank was isolated from the external surrounding. Insulating material (glass wool) was used in the tank unit. A heater with a power rating of 2.5 kW was employed inside the feed tank to maintain the required temperature. A Pt-100 temperature sensor was also attached to the heater to measure a wide temperature range. Autotuning temperature controller called TC513BX was equipped to maintain the temperature. The membrane holder was made of AISI 304-grade stainless steel with a surface area of 15 cm^2^. A circulation pump with adjustable speed was equipped on the feed side. A vacuum of 31.325 kPa was kept on the permeate side using a vacuum pump equipped with a pressure gauge (PN2299 pressor sensor with display). The pressure gauge was also equipped with a pressure sensor to maintain the system pressure constant. The stirrer was fixed in the feed container to keep the same temperature throughout the tank.

After the membrane reached equilibrium, a fixed vacuum was induced, and on the permeate side, the vapors were accumulated after condensation in the cold traps by inserting liquid nitrogen at uniform intervals. Subsequently, permeate weight was measured by using a digital microbalance, and the permeation flux was calculated. KAFI smart Karl Fischer Titrator measured the composition of the permeate. A minimum of 3 different readings were taken, and the mean value was considered for parameters such as permeation flux, selectivity and PSI. The PV performance of the fabricated hybrid membranes was analyzed by calculating the total permeation flux, separation selectivity and pervaporation-separation index (PSI) using the equations reported in the literature [16,53,54,55,56,57].

## Figures and Tables

**Table 1:** Permeation flux, separation selectivity and permeance data of the developed membranes at various temperatures.

Membrane	Temperature (°C)	*J* (kg/m^2^ h)	α_Sep_	*P_i_/l* (GPU)
M	30	0.0844 ± 0.0022	1557.67 ± 11.68	1688.00
	40	0.0891 ± 0.0015	1412.00 ± 8.00	1781.33
	50	0.0922 + 0.0010	1378.67 + 6.11	1843.33
M-1	30	0.0949 ± 0.0008	2238.33 ± 7.64	1897.33
	40	0.1030 ± 0.0010	2028.33 ± 6.51	2060.00
	50	0.1078 ± 0.0003	1912.00 ± 4.00	2155.33
M-2	30	0.1060 ± 0.0005	2425.67 ± 6.03	2077.28
	40	0.1083 ± 0.0005	2192.00 ± 5.29	2123.53
	50	0.1134 ± 0.0004	2045.67 ± 6.03	2222.88
M-3	30	0.1152 ± 0.0004	2807.33 ± 6.43	2259.48
	40	0.1211 ± 0.0005	2407.67 ± 7.64	2374.51
	50	0.1285 ± 0.0003	2258.00 ± 4.00	2519.61
M-4	30	0.1240 ± 0.0020	3612.33 ± 6.03	2384.62
	40	0.1310 ± 0.0036	2937.00 ± 6.24	2519.23
	50	0.1333 ± 0.0050	2449.67 ± 7.09	2564.10

GPU = gas permeation unit = 10^−6^ cc (STP)/cm^2^/s/cm Hg.

**Table 2:** Analysis of variance for total permeation flux and selectivity vs. membrane type and temperature.

Source of Variation	Degree of Freedom (df)	Sum of Squares (SS)	Mean Squares (MS)	F	*p*-Value	F _Crit._
**Total Permeation Flux**						
Type of Membrane (M)	4	0.009358	0.002339	659.0334	9.29 × 10^−29^	2.689628
Temperature(T)	2	0.000773	0.000386	108.8769	1.76 × 10^−14^	3.31583
Interaction (M × T)	8	6.66 × 10^−5^	8.33 × 10^−6^	2.346156	0.043261	2.266163
Error	30	0.000106	3.55 × 10^−6^			
**Selectivity**						
Type of Membrane (M)	4	11,681,129	2,920,282	62,906.99	2.23 × 10^−58^	2.689628
Temperature (T)	2	2,077,327	1,038,663	22,374.27	2.46 × 10^−48^	3.31583
Interaction (M × T)	8	890,847.7	111,356	2398.764	6.46 × 10^−40^	2.266163
*Error*	30	1392.667	46.42222			

## Data Availability

The study did not report any data.
